# Integrated analyses of brain and platelet omics reveal their common altered and driven molecules in Alzheimer's disease

**DOI:** 10.1002/mco2.180

**Published:** 2022-10-13

**Authors:** Haitao Yu, Mengzhu Li, Qihang Pan, Yanchao Liu, Yao Zhang, Ting He, Huisheng Yang, Yue Xiao, Ying Weng, Yang Gao, Dan Ke, Gaoshang Chai, Jian‐Zhi Wang

**Affiliations:** ^1^ Department of Pathophysiology School of Basic Medicine Key Laboratory of Education Ministry of China/Hubei Province for Neurological Disorders Tongji Medical College Huazhong University of Science and Technology Wuhan China; ^2^ Department of Basic Medicine Wuxi School of Medicine Jiangnan University Wuxi Jiangsu China; ^3^ Department of Neurosurgery Wuhan Central Hospital Affiliated to Tongji Medical College Huazhong University of Science and Technology Wuhan China; ^4^ Department of Neurosurgery Tongji Hospital Tongji Medical College Huazhong University of Science and Technology Wuhan China; ^5^ Department of Endocrinology Liyuan Hospital Tongji Medical College Huazhong University of Science and Technology Wuhan China; ^6^ Co‐Innovation Center of Neuroregeneration Nantong University Nantong China; ^7^ Institute of Acupuncture and Moxibustion China Academy of Chinese Medical Sciences Beijing China

**Keywords:** Alzheimer's disease, brain tissue, omics, peripheral biomarkers, platelet

## Abstract

Platelets may serve as a perfect peripheral source for exploring diagnostic biomarkers for Alzheimer's disease (AD); however, the molecular linkage between platelet and the brain is missing. To find the common altered and driving molecules in both brain and the platelet, we performed an integrated analysis of our platelet omics and brain omics reported in the literature, and analyzed their correlations with AD‐specific pathology and cognitive impairment. By integrating the gene and protein expression profiles from 269 AD patients, we deduced 239 differentially expressed proteins (DEPs) appeared in both brain and the platelet, and 70.3% of them had consistent changes. Further analysis demonstrated that the altered brain and peripheral regulations were pinpointed into 10 imbalanced pathways. We also found that 117 DEPs, including ADAM10, were closely associated to the AD‐specific β‐amyloid and tau pathologies; and the changes of IDH3B and RTN1 had a potential diagnostic value for cognitive impairment analyzed by machine learning. Finally, we identified that HMOX2 and SERPINA3 could serve as driving molecules in neurodegeneration, and they were increased and decreased in AD patients, respectively. Together, this integrated brain and platelet omics provides a valuable resource for establishing efficient peripheral diagnostic biomarkers and potential therapeutic targets for AD.

## Graphical abstract

By employing multiple statistical analyses plus machine learning, we identified and validated multiple deregulated central and peripheral network in AD patients, which provided rich resources for understanding the pathogenesis of AD and large‐scale biomarker validation. We also highlighted the key‐driven role of HMOX2 and SERPINA3 in the AD pathogenesis, and the potential diagnostic value of RTN1 and IDH3B for AD.

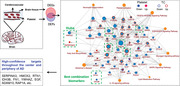

## 1 INTRODUCTION

Alzheimer's disease (AD) is the most common form of dementia characterized pathologically by accumulation of β‐amyloid (Aβ) and hyperphosphorylated tau proteins.^1^, ^2^ However, the current therapeutic studies by targeting Aβ plaques and tau have been challenged,^3^ suggesting the involvement of complex mechanisms in AD. In addition, one of the bottlenecks leading to the failed drug development is the lack of effective and non‐brain invasive biomarkers for the early diagnosis of AD.^3^ Large‐scale, comprehensive, and impartial molecular analysis of AD patients is particularly important to identify the complex mechanisms and potentially effective biomarkers involved in the pathogenesis of AD, with priority given to the association with clinical cognitive decline.

Integrated analysis of large, multi‐omics datasets is an effective way to identify key molecular pathways and potential drug targets.^4^, ^5^ In brain tissue, previous studies have integrated multiple genomic datasets including four brain regions, entorhinal cortex (EC), hippocampus (HP), temporal cortex (TC), and frontal cortex (FC), to fully unravel the central pathological regulatory networks and driver genes of AD.^6^ Considering the similarities between neuron and platelet biology, platelets may be a good peripheral source for exploring AD diagnostic biomarkers.^7^ Our latest Huazhong University of Science and Technology (HUST) platelet proteomics reveals a comprehensive subnetwork of cognitive decline in the elderly and a set of potential biomarkers for the early diagnosis of AD.^8^ Studies have shown potential dysregulation pathways in AD from different perspectives,^9^, ^10^ but the integrated analysis of central and peripheral omics data related to AD is still missing. This type of analysis is particularly important for our understanding to the complex pathological mechanisms of AD and thus to explore reliable peripheral diagnostic biomarkers.

Here, we integrated ultra‐deep platelet proteomics with multiple brain regions to reflect the central and peripheral links during AD. By integrating brain and platelet data, we identified the disordered networks highly associated with AD, such as platelet activation, interleukin‐18 (IL‐18) signaling pathway, and epidermal growth factor/epidermal growth factor receptor (EGF/EGFR) signaling pathway, by which high‐confidence targets throughout the center and periphery of AD were deduced. By co‐expression analysis of the associated omics with the patients’ clinical cognitive decline modules, the role of changed heme oxygenase 2 (HMOX2) and SERPINA3 was highlighted in cognitive impairment. Multiple algorithms demonstrated the diagnostic value of IDH3B and RTN1 for AD.

## 2 RESULTS

### 2.1 Global characterization of brain and platelet expression profiles of AD patients

**FIGURE 1:**
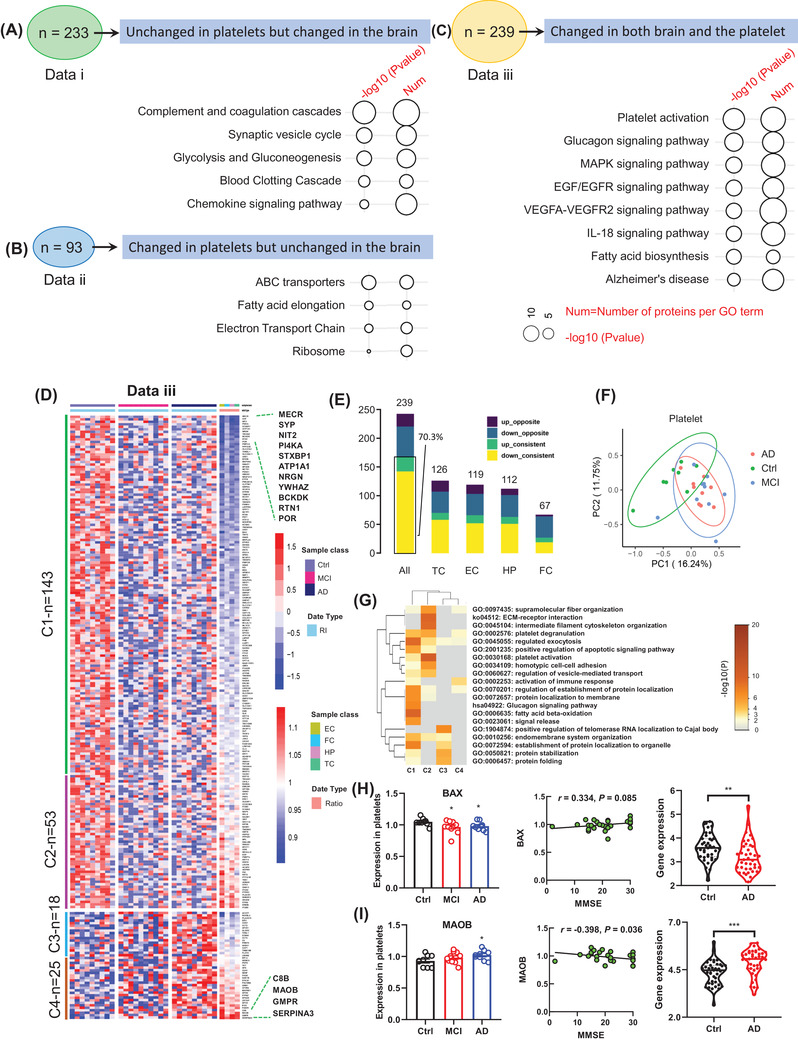
Changed pathways and dysregulated molecules in brain and platelet of Alzheimer's disease (AD) patients. To fully understand the central and peripheral dysregulated pathways, we performed pathway enrichment analysis on the molecules of the three modules: (A) unchanged in platelets but changed in the brain (data i); (B) changed in platelets but unchanged in the brain (data ii); (C) changed in both brain and the platelet (data iii). (D) The heat map shows the differential molecular expression profile of central and platelets (data iii). All 239 differentially expressed molecules were divided into four clusters (C1, C2, C3, C4). (E) A statistical summary of 239 differentially expressed molecules in brain regions and platelets. (F) Principal component analysis (PCA) confirmed the efficiency of differential proteins in platelets. (G) The four clusters of different molecules are enriched in different biological processes. The ‐log10(*P*) was used to define the enrichment strength of biological processes. (H and I) Expression of representative high‐risk genes BAX and MAOB in platelets and entorhinal cortex, and their correlation with Mini‐Mental State Examination (MMSE). Stars represent significant correlations: ^*^ *p* < 0.05; ^**^ *p* < 0.01; ^***^ *p* < 0.001. EC, entorhinal cortex; FC, frontal cortex; HP, hippocampus; IL, interleukin; MCI, mild cognitive impairment; TC, temporal cortex

By comparing AlzData datasets, we found that 91.8% of platelet proteins (2696 proteins in AlzData) were also expressed in the four brain regions, and 8.2% of platelet proteins were peripheral unique (Figure S1). Furthermore, 72.5% of the proteins were highly conserved in brain and platelet without significant changes (Figure S1), while 7.9% of the proteins (data i) were significantly changed in the brain but not in the platelet (Figure S1). The main enriched pathways were complement and coagulation cascades, synaptic vesicle cycle, glycolysis and gluconeogenesis, blood clotting cascade, and chemokine signaling pathway (Figure 1A). In addition, we found that 3.2% of the proteins (data ii) were significantly changed in the platelet but not in the brain, and the main enriched pathways were ABC transporters, fatty acid elongation, electron transport chain, and ribosome (Figure 1B).

Surprisingly, 239 of the 360 differentially expressed proteins (DEPs) in platelets (data iii) were also differentially expressed in the brain (Figures S1 and 1D), of which 168 (70.3%) DEPs showed a consistent trend of change between brain and platelets (Figure 1E), and the main enriched pathways were platelet activation, glucagon signaling pathway, mitogen‐activated protein kinases (MAPK) signaling pathway, EGF/EGFR signaling pathway, vascular endothelial growth factor A–vascular endothelial growth factor receptor 2 (VEGFA–VEGFR2) signaling pathway, IL‐18 signaling pathway, fatty acid biosynthesis, and AD (Figure 1C). Specifically, 126, 119, 112, and 67 DEPs identified in TC, EC, HP, and FC regions of the brain were also significantly changed in the platelet (Figure 1E). By principal component analysis (PCA) analysis, 239 DEPs could effectively distinguish cognitive impairment from non‐cognitive impairment (Figure 1F), though the changes of these 239 DEPs could not distinguish mild cognitive impairment (MCI) from AD groups. These data further support the diagnostic value of platelet in AD.

According to the central and peripheral consistence, we further divided the 239 DEPs into four clusters: cluster 1 (C1) contains 143 uniformly decreased molecules, cluster 2 (C2) contains 53 platelet‐decreased but brain‐increased molecules, cluster 3 (C3) contains 25 uniformly increased molecules, and cluster 4 (C4) contains 18 platelet moderately increased but brain‐decreased molecules (Figure 1D,E). Many AD‐related molecules were on the top‐altered proteins, that is, the most altered molecules in clusters 1 and 4, such as synaptophysin (SYP), 14‐3‐3 protein zeta/delta (YWHAZ), reticulon‐1 (RTN1), and complement component C8 beta chain (C8B) (Figure 1D). Gene ontology (GO) pathway analysis showed that these DEPs were enriched in multiple dysregulated pathways, such as platelet degranulation, regulated exocytosis, platelet activation, regulation of vesicle‐mediated transport, fatty acid beta‐oxidation, positive regulation of telomerase RNA localization to Cajal body, protein stabilization, and protein folding (Figure 1G). We also found that the high‐risk genes (ref: https://www.disgenet.org/home/), such as BAX^11^ and MOAB,^12^ were respectively, decreased and increased in EC and platelet, and the changes were slightly correlated with Mini‐Mental State Examination (MMSE) (BAX: *r* = 0.334, *p* = 0.085; MAOB: *r* = 0.398, *p* = 0.036; Figure 1H,I).

### 2.2 Machine learning establishes efficient diagnostic models for AD

**FIGURE 2:**
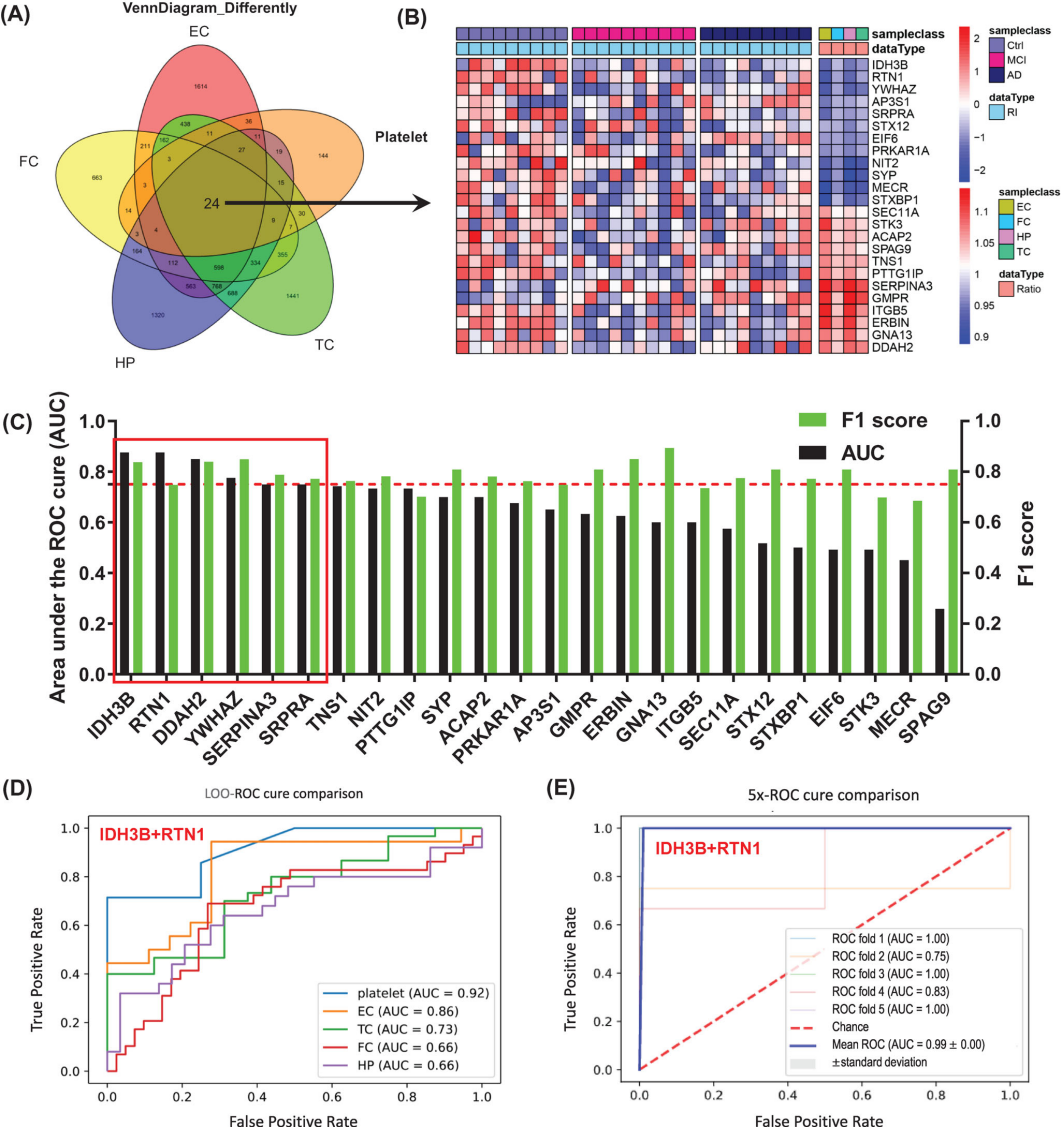
Machine learning identifies biomarkers that can effectively distinguish cognitive impairment from normal cognitive population. (A) Venn analysis of differential molecules in four brain regions and platelets. (B) Twenty‐four molecules were significantly differentially expressed in all four brain regions and platelets. (C) Twenty‐four co‐differentially expressed molecules were modeled and analyzed by the leave‐one‐out (LOO) algorithm, and the corresponding area under the curve (AUC) and F1 scores were obtained, according to AUC >0.75 and F1 score >0.7, six candidate biomarkers were selected. The red boxes are the six candidate proteins. (D) Based on the LOO algorithm, the under the receiver operating characteristic curve (ROC) for combination biomarkers (IDH3B + RTN1) in platelets and different brain regions. (E) Based on the fivefold cross‐validation, the under the ROC for combination biomarkers (IDH3B + RTN1) in platelets. AUC was based on true‐positive rate and false‐positive rate: true‐positive rate = [true positive/(true positive + false negative)]; false‐positive rate = [false positive/(true negative + false negative)]; precision = [true positive/(true positive + false positive)]; recall = [true positive/(true positive + false negative)]. In addition, F1 score = 2 × (precision × recall)/(precision + recall). AD, Alzheimer's disease; EC, entorhinal cortex; FC, frontal cortex; HP, hippocampus; MCI, mild cognitive impairment; TC, temporal cortex

Notably, 24 molecules were significantly dysregulated in all four brain regions and platelets, providing good clues for further exploration of diagnostic biomarkers or potential therapeutic targets (Figure 2A,B). The increased candidate proteins include (1) alpha‐1‐antichymotrypsin (ACT/SERPINA3), an acute phase serum glycoprotein involved in complement and inflammatory pathways as a serine protease inhibitor,^13^ and it can promote Aβ polymerization.^14^ (2) Guanosine monophosphate reductase, a regulator in AMP‐activated protein kinase (AMPK) and adenosine receptor pathways, involved in AD‐like tau hyperphosphorylation^15^ (Figure 2B). The significantly decreased candidate proteins in both central and platelets include (1) isocitrate dehydrogenase (NAD) subunit beta (IDH3B), an enzyme that can catalyze isocitrate decarboxylation to form alpha‐ketoglutarate in tricarboxylic acid cycle (TCA), a significant reduction of IDH3B was also reported previously in cerebrospinal fluid and blood.^16^ (2) RTN1, a protein co‐immunoprecipitated with BACE1 and can inhibit BACE1 activity.^17^ (3) YWHAZ, a highly conserved chaperone molecule involved in cell signal transduction, cell cycle regulation, transcription, etc., and the level of 14‐3‐3 is decreased in the AD brains (Figure 2B).

By using machine learning, that is, the leave‐one‐out method, we constructed diagnostic models using the above identified 24 DEPs from four AD brain regions and the peripheral platelet with data in platelet (Figure 2C). Six proteins (IDH3B, RTN1, SRPRA, YWHAZ, SERPINA3, and DDAH2) with top discriminating power were identified with area under the curve (AUC) >0.75 and F1 score >0.7 (Figure 2C), among which platelet IDH3B showed the strongest diagnostic value with AUC of 0.88 and accuracy of 0.75 (Figure 2C). By further maximizing permutation analyses for the above six molecules, we found that the platelet IDH3B and RTN were the best combination for identifying cognitive decline with AUC of 0.92, accuracy of 0.82, F1 score of 0.87, recall of 0.90, and precision of 0.87 (Figures 2D and S2A). In central system, IDH3B and RTN1 also showed strong diagnostic efficiency in EC subset with an AUC of 0.86 and an accuracy of 0.82 (Figures 2D and S2B). Further fivefold cross‐validation results also showed that combination of IDH3B and RTN1 had good diagnostic efficacy with an average AUC of 0.99 (Figure 2E). Additionally, the expression level of IDH3B and RTN1 was consistently decreased in the AD brains and the platelet (Figure S3A,C,E,F). Correlation analysis showed that IDH3B and RTN1 were moderately correlated with MMSE (Figure S3B,D).

### 2.3 The network linkage analyses reveal AD pathology‐correlated brain and platelet molecules

**FIGURE 3:**
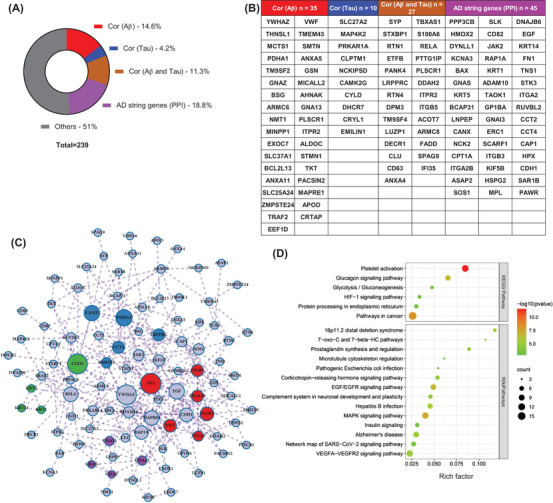
High‐rank differentially expressed proteins (DEPs) and the complex linkage network correlated to Alzheimer's disease (AD) like β‐amyloid (Aβ) and tau pathologies in brain and platelet. (A) Pie chart showing the proportion of molecules correlated with Aβ, tau, or Aβ and tau. (B) Specific molecules correlated with Aβ, tau, or Aβ and tau. (C) A regulatory network of AD pathologically related differential molecules, and the size of the circle indicates its importance in the pathogenic network. (D) Kyoto Encyclopedia of Genes and Genomes (KEGG) and wiki pathway enriched in AD pathology‐related proteins. The *X*‐axis represents the rich factor, the bubble size represents the number of targets enriched in terms, and the color indicates the *p*‐value

To characterize the association of the dysregulated molecules with the AD‐specific Aβ and tau pathologies, we referred to genome‐wide gene expression analyses and databases related to the development of amyloid or tau pathology,^18^ which were also applied in the AlzData dataset. Surprisingly, a total of 117 (49% in the data iii) DEPs were strongly associated with the AD pathologies, further reinforcing the value of platelet in developing biomarkers for the early diagnosis of AD (Figure 3A). Specifically, Aβ‐related molecules accounted for 14.6% (*n* = 35, i.e., YWHAZ, GSN), tau‐related molecules accounted for 4.2% (*n* = 10, i.e., PRKAR1A), both Aβ‐ and tau‐related molecules accounted for 11.3% (*n* = 27, i.e., RTN1/4, CLU, S100A6), and AD string genes [protein–protein interaction (PPI)] accounted for 18.8% (*n* = 45, i.e., PPP3CB, BAX, ADAM10) (Figure 3A,B).

By integrating all 117 AD‐specific Aβ‐ and tau‐related molecules, a complete central and peripheral regulatory network reflecting AD pathogenesis was constructed (Figure 3C). By which, FN1, YWHAZ, EGF, ADAM10, HMOX2, CANX, CYLD, PDHA1, RAP1A could be the pivotal molecules in this complex network, which deserves further investigation. Further GO enrichment analysis revealed that AD pathology‐related differential molecules were mainly involved in platelet activation, VEGFA−VEGFR2 signaling pathway, AD, MAPK signaling pathway, glucagon signaling pathway, and biological processes such as blood coagulation, cell components such as focal adhesion, and molecular functions such as calcium ion binding (Figure 3D).

### 2.4 A comprehensive analysis identifies hub molecules and platelet biomarkers for AD‐like cognitive impairment

To explore the driving molecules for changed central or peripheral networks in AD, we adopted weighted correlation network analysis (WGCNA) to analyze the relationships of the subjects' common diseases, including age, sex, MMSE score, basic diseases such as hypertension and coronary heart disease, AD pathology such as Aβ, high‐risk gene APOE, with DEPs observed by brain and platelet omics.

**FIGURE 4:**
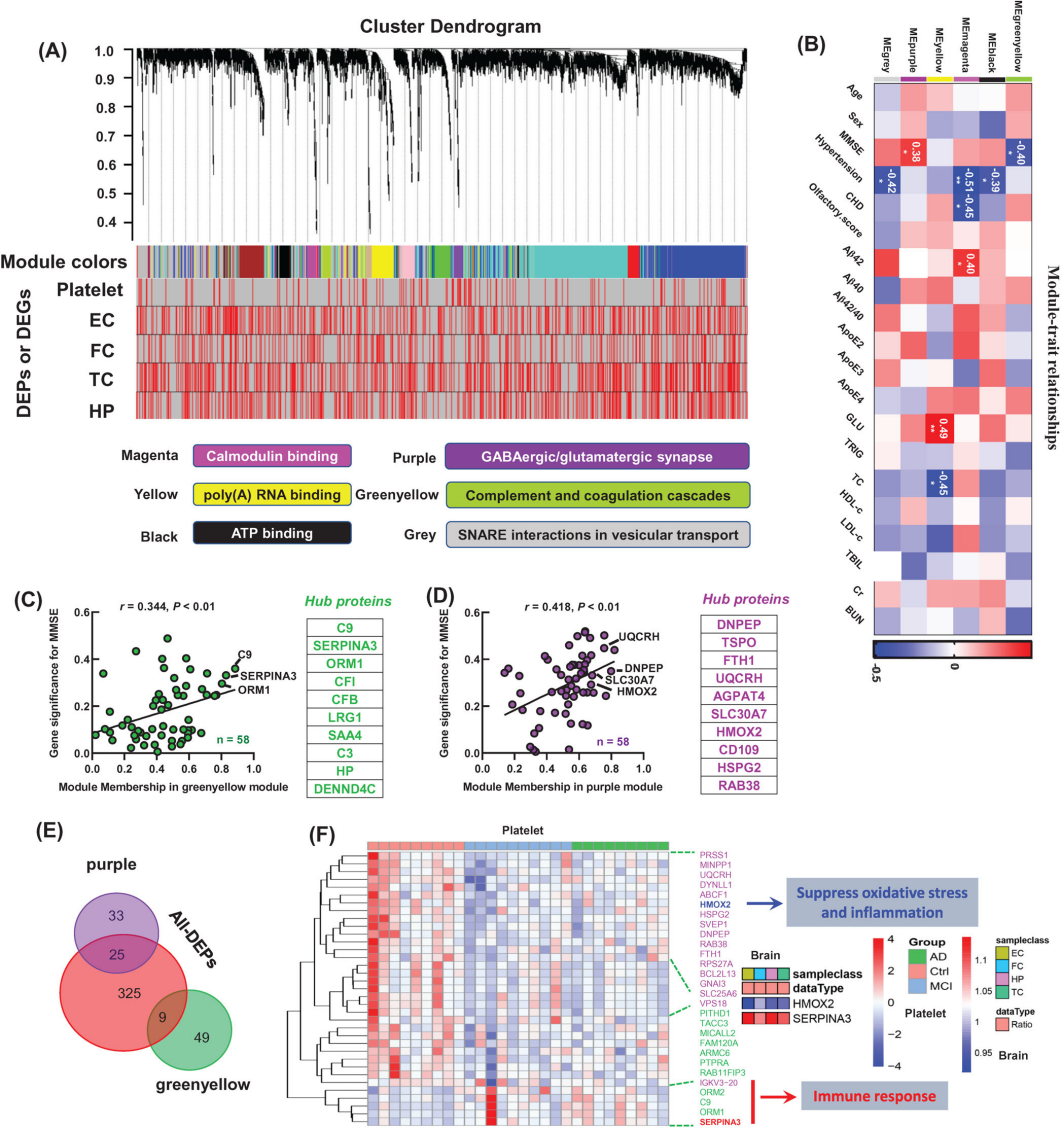
Upstream driving regulators of cognitive impairment revealed by weighted correlation network analysis (WGCNA). (A) WGCNA cluster dendrogram generated by unsupervised hierarchical clustering of all proteins in the entire platelet proteomic data set based on topological overlap, followed by branch cutting, revealing the network modules coded by different colors. The genes corresponding to different brain regions were matched with platelet proteome data in columns 2–5, with red representing differentially expressed molecules. Functional categories enriched by representative modules. (B) The correlation between module eigengenes (MEs) and clinical phenotype, such as age, sex, Mini‐Mental State Examination (MMSE) score, hypertension. The values in the heatmap are Pearson's correlation coefficients. Stars represent significant correlations: ^*^ *p* < 0.05; ^**^ *p* < 0.01. (C) The proteins with significantly reduced expression in mild cognitive impairment (MCI) and Alzheimer's disease (AD) were significantly positively correlated with MMSE and top hub proteins. (D) The proteins with significantly reduced expression in MCI and AD were significantly positively correlated with MMSE and top hub proteins. (E) Venn analysis of differentially expressed proteins (DEPs) and MEpurple or MEgreenyellow modules showed that the MEpurple module had 25 DEPs and the MEgreenyellow module had nine DEPs. (F) Differential expression heat map of representative molecules in MEpurple or Megreenyellow modules. DEG, differentially expressed gene; EC, entorhinal cortex; FC, frontal cortex; HP, hippocampus; TC, temporal cortex

Integrating the AlzData database, including gene sets of four brain regions: EC, HP, TC, and FC, the 2994 previously identified platelet proteins^8^ were divided into modules by the module eigengene (ME) (Figure 4A, red in the heat map represents DEPs or differentially expressed genes). We observed that six modules, that is, calmodulin binding (MEmagenta), poly(A) RNA binding (MEyellow), adenosine 5'‐triphosphate (ATP) binding (MEblack), GABAergic/glutamatergic synapse (MEpurple), complement and coagulation cascades (MEgreenyellow), and SNARE interactions in vesicular transport (MEgrey), were significantly correlated to the external information, such as age, sex, MMSE scores, hypertension, etc. (Figure 4B). The MMSE score was significantly correlated with MEgreenyellow (*r* = ‐0.40, *p* < 0.05) and MEpurple (*r* = 0.38, *p* < 0.05) (Figure 4B). Gene significance for MMSE was significantly correlated with greenyellow (*r* = 0.344, *p* < 0.01) and purple modules (*r* = 0.418, *p* < 0.01), respectively (Figure 4C,D), and HMOX2, UQCRH, C9, and SERPINA3 were identified as hub proteins (Figure 4C,D).

By Venn logic analysis to the 239 DEPs, we found that the decreased HMOX2 and increased SERPINA3 in platelet (in the MEpurple and MEgreenyellow modules, Figure 4E) showed favorable consistency in different brain regions (Figure 4F). Biochemical experiments and integration analysis verified that the decreased HMOX2 and increased SERPINA3 could be hub molecules, show showed good consistency in the central and peripheral systems of AD patients and the AD transgenic mice, and was consistent with the proteomics results (Figures S4 and S5). SERPINA3 was increased in the HC of 5×FAD^19^ and hTau mice mined from our previous proteomic datasets (Figure S5G,H).^20^

### 2.5 Integrated bioinformatics and machine learning establishes high‐confidence targets and models for AD diagnosis and drug development

**FIGURE 5:**
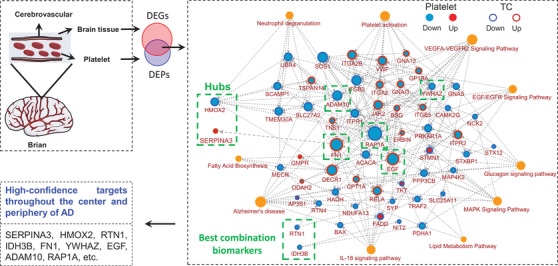
The altered brain and platelet key proteins and their network linkage in Alzheimer's disease (AD) patients. Protein–protein interaction (PPI) network was constructed by integrating key dysregulated pathways and related proteins (increased molecular: red; decreased molecular: blue; high‐confidence targets throughout the center and periphery of AD: green). DEG, differentially expressed gene; DEP, differentially expressed protein; TC, temporal cortex

By gene network function and molecular integration analyses, we classified the DEPs into the following 10 clusters: AD, platelet activation, neutrophil degranulation, VEGFA–VEGFR2 signaling pathway, EGF/EGFR signaling pathway, lipid metabolism pathway, fatty acid biosynthesis, glucagon signaling pathway, MAPK signaling pathway, and IL‐18 signaling pathway (Figure 5, red represents increased, blue represents decreased, and the inner and outer circles represent platelets and TC brain regions), all of which were closely related to neurological diseases including AD.^21^, ^22^, ^23^, ^24^, ^25^, ^26^

We also observed that the AD‐related molecules, including ADAM10, PPP3CB, RTN4, BAX, ITPR1, ITPR2, NDUFA13, FADD, RELA, TRAF2 and MAP4K2, SOS1, and ERBIN, were the most significantly enriched nodes (Figure 5). ADAM10 showed the highest connectivity in the whole pathway and a decreased consistency in the central and peripheral during the cognitive impairment process, indicating its important role in AD.

Together, the comprehensive integrative analysis of platelet and brain omics data demonstrated that the molecular changes in platelet can well reflect the pathological mechanism of the brain in AD patients, which provides high‐confidence platelet targets for future large‐scale validation in AD‐related population (Figure 5), highlighting the key regulator role of HMOX2 and SERPINA3 in AD, as well as the potential diagnostic value of RTN1 and IDH3B.

## 3 DISCUSSION

AD is an irreversible and devastating neurodegenerative disease, and with the aging of the population, the impact is becoming more and more serious. Therefore, the development of convenient and stable peripheral biomarkers for AD early diagnosis is greatly needed.

With advances in proteomics, large‐scale protein expression profiles of brain and peripheral biological fluids have revealed complex molecular mechanisms and effective diagnostic models for the progression of AD. Bai et al.,^23^ through the combination of Aβ and tau pathological depth in AD human brain tissue, revealed A wide range of protein differential expression and significant dysregulation of 17 pathways in the progression of AD. Matthias Mann's team identified more than 20 cerebrospinal fluid proteins and a series of potential candidate biomarkers including tau, SOD1, PARK7, and YKL‐40 that were associated with AD pathology in three independent cohorts.^10^ Recently, Ip's team identified 429 AD‐related dysregulated plasma proteins, creating a highly efficient diagnostic model.^27^ In addition, proteomics identified ANXA5, VGF, GPM6A, and ACTZ are new signature proteins in AD extracellular vesicles.^28^ However, with the innovation of technology, the comprehensive and in‐depth disclosure of platelet expression profile seems to be neglected. In fact, compared with cerebrospinal fluid and plasma, platelets are more stable, and have many neuron‐like biological characteristics, which are ideal source for discovering biomarkers for early diagnosis of AD.^7^ In this study, we used tandem mass tags (TMT) tags combined with liquid chromatography—mass spectrometry/mass spectrometry technology to comprehensively outline the platelet protein expression profile during cognitive impairment and integrate them with brain gene expression profile data for analysis, further WGCNA and subsequent bioinformatics analysis, the protein connection network, related pathological modules and specific cell type positioning were fully characterized, reflecting the potential linkage mechanism between central and peripheral in AD. Scientists have also used different sources of samples from different persons for the analysis,^16^, ^29^ exactly as has done in our current study. By this type of integrated analysis, we have revealed molecular connections between brain and platelet, which is important not only for further in‐depth mechanism studies, but also for the diagnosis of AD by using platelet samples.

Firstly, integrating all 360 differential platelet proteins, we found that 239 of them were also differentially expressed in the central system, and 70.3% of them showed central and peripheral consistency, suggesting that AD may be a systemic molecular disorder rather than a simple brain omics case. Due to the depth of platelet proteome, we can construct the linkage network between central and peripheral systems in the progression of AD including 10 main pathways. Consistent with our previous analysis, this further highlights the systemic synergistic role of AD, platelet activation, and lipid metabolism pathways in AD.^8^ Excitingly, we identified 117 proteins that may drive the progression of AD through enrichment analysis of Aβ, tau pathology, and AD high‐risk genes. PPI analysis highlighted the key regulatory roles of FN1, YWHAZ, EGF, ADAM10, HMOX2, CANX, CYLD, PDHA1, and RAP1A in AD pathology. In fact, FN1 had been shown in our previous analysis to play a role in platelet activation and has a greater diagnostic value, which was positively correlated with MMSE.^8^ YWHAZ, a member of the 14‐3‐3 protein family, is involved in the regulation of brain neural development and signal transduction,^30^ while ADAM10 is associated with Aβ, the hallmark pathology of AD, which are also considered to have great application value in the clinical diagnosis of dementia.^31^, ^32^ PDHA1, pyruvate dehydrogenase E1 component subunit alpha, is a key component of glucose metabolism, and its deficiency can lead to lactic acid accumulation and impair learning and memory in mice.^33^ Interestingly, our data showed that it was significantly reduced in the central and peripherally in patients with AD.

Based on the existing 24 dysregulated molecules in four brain regions and platelets, we constructed models for central and peripheral diagnosis. We found that the combined biomarkers of IDH3B and RTN1 could well identify people with or without cognitive impairment, whether it was peripheral platelets or central EC, which gave me great confidence that peripheral lamina molecules could indeed reflect the changes of central pathological mechanism. Interestingly, IDH3B, isocitrate dehydrogenase (NAD) subunit beta, primarily catalyzing the decarboxylation of isocitrate into alpha‐ketoglutarate in the TCA.^34^ TCA was a very important link in glucose metabolism,^35^ and fluorodeoxyglucose‐positron emission tomography was a marker of specificity and sensitivity to identify MCI.^36^ The expression changes of IDH3B may be an alternative way to detect brain glucose metabolism disorders in AD patients. RTN1 inhibits amyloid precursor protein processing by blocking BACE1 activity,^37^ and it was significantly reduced in the brains of AD patients,^38^ which was consistent with our results. RTN1 is a biomarker worthy of attention, and its reduction may contribute to the early pathologic formation of AD, namely Aβ deposition.^17^

Secondly, based on the WGCNA analysis, we revealed the MMSE‐related modules at the overall level of the proteome, highlighting the hub genes of the HMOX2 and SERPINA3, which are consistent with the trend in central brain regions, and significantly decreased and increased in AD. HMOX2 is a key rate‐limiting enzyme in heme metabolism, promoting the decomposition of heme into CO, ferrous ions, and biliverin, which are involved in many physiological processes.^39^ The deficiency of HMOX2 leads to iron metabolism obstacle, causing iron deposition.^40^ Ferrous ions themselves can also participate in subsequent redox reactions.^40^, ^41^ Therefore, HMOX2 deficiency is also associated with the production of reactive oxygen species and inflammatory factors,^40^, ^42^ and HMOX2 has a certain neuroprotective effect on cerebral hemorrhage injury.^42^ In addition, the database of over one million people reveals that heme metabolism may be the key to health and life expectancy.^43^ Furthermore, the complement inflammation‐related molecule SERPINA3 is increased in AD and promotes Aβ aggregation and amyloid production, which has been widely reported.^14^, ^44^, ^45^ Interestingly, a recent study showed that APOE4 specifically upregulates ACT levels in the brain and promotes microglia activation in aging mice.^46^

## 4 CONCLUSIONS

For the first time, the linkage network between AD brain and platelets was constructed, providing rich resources for understanding the pathogenesis of AD and large‐scale biomarker validation. In addition, highlighting the key regulator role of HMOX2 and SERPINA3 in AD, as well as the potential diagnostic value of RTN1 and IDH3B. Overall, the integration of central and peripheral omics is novel, reflecting the huge application potential of proteomics‐driven precision medicine in AD.

The main limitations of the current study are as follows: the brain tissue and platelets samples were obtained from different AD subjects, only a small sample size was used for the proteomic analysis to minimize the impact on the accuracy of proteomics data, which could be improved in future studies.

## 5 MATERIALS AND METHODS

### 5.1 Study design

To find reliable periphery biomarkers and hub molecules for AD diagnosis and therapeutic targets, we performed an integrated analysis to our platelet omics dataset^8^ with the brain region‐specific omics datasets from AlzData database (http://www.alzdata.org/).^6^ In our recent high‐throughput mass spectrometry analysis of the HUST peripheral platelet proteome dataset, a total of 4165 proteins were identified, which has been the most in‐depth platelet proteomics on cognitive decline providing a wealth of data for the systematic study of potential biomarkers of AD. It should be emphasized that recruitment of the 28 Han people (10 MCI, 9 AD, and 9 Ctrl) applied in the platelet proteome dataset was based on the MMSE score^47^ and National Institute on Aging and the Alzheimer's Association Guidelines,^48^ with consideration of AD high‐risk genes and the elderly metabolic diseases and exclusion of other mental disorders, such as brain trauma, schizophrenia, etc.

The AlzData has 20 Genome Sequencer Enhanced (GSE) series dataset, including 540 human postmortem brain tissues (AD = 269, Ctrl = 271), four brain regions (EC: 39 vs. 39, HP: 74 vs. 65, TC: 52 vs. 39, FC: 104 vs. 128), and thousands of gene expression data through cross‐platform standardization, providing valuable resources for understanding the pathological mechanism of AD, searching for potential drug targets, and early diagnosis of biomarkers.

Platelet proteomics dataset was normalized by Perseus platform, which contains a comprehensive portfolio of statistical tools for high‐dimensional omics data analysis covering normalization, pattern recognition, time‐series analysis, cross‐omics comparisons, and multiple‐hypothesis testing.^49^ And multiple datasets of brain genomics in AlzData have been already normalized.^6^ Specifically, all processed expression data from the same brain region were merged by algorithm ComBat in R package inSilicoMerging.^50^, ^51^

**FIGURE 6:**
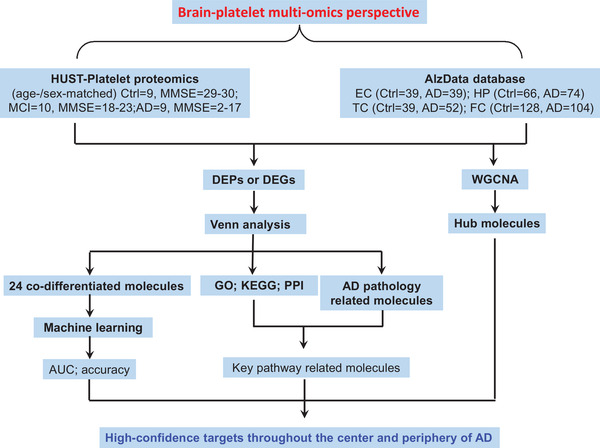
Rationale and workflow of the present study. A systematic transformation pipeline, including bioinformatics analyses, such as weighted correlation network analysis (WGCNA), Venn analysis, Kyoto Encyclopedia of Genes and Genomes (KEGG), gene ontology (GO), and protein– interaction network (PPI), and machine learning, was constructed to integrate the central and peripheral multiple omics data. Using this platform, the central–peripheral linkage network, the hub molecules, and candidate diagnostic biomarkers were identified. AD, Alzheimer's disease; AUC, area under the curve; DEG, differentially expressed gene; DEP, differentially expressed protein; EC, entorhinal cortex; FC, frontal cortex; HP, hippocampus; HUST, Huazhong University of Science and Technology; MCI, mild cognitive impairment; MMSE, Mini‐Mental State Examination; TC, temporal cortex

To integrate platelet omics with brain datasets and disease phenotypes, we used WGCNA, differential molecular analysis, Venn analysis, Kyoto Encyclopedia of Genes and Genomes (KEGG), and PPI network analysis (Figure 6), by which the common altered brain and platelet molecules closely associated with cognitive impairment, and multiple driving genes and potential biomarkers for cognitive decline were revealed.

### 5.2 Animals

The 5×FAD [B6.Cg‐Tg(APPSwFlLon,PSEN1*M146L*L286V)6799V] mice (male, 28–32 g, 6/8/10‐month, *n* = 7) and control mice (wild‐type, male, 28–32 g, 6/8/10‐month, *n* = 6) were obtained from the Jackson Laboratory (Maine, USA). 3×Tg [stock no: 34830, 129S4.CgTg(APPSwe,tauP301L)1LfaPsen1tm1Mpm/Mmjax] mice (male, 32 g, 7‐month) and P301L (male, 24–32 g, 3/6/12‐month, *n* = 8–9) and wild‐type mice (male, 24–32 g, 3/6/12‐month, *n* = 7–8) were a gift from Prof. Xifei Yang (Shenzhen Center for Disease Control and Prevention). All animals were housed in a 12‐h–12‐h light–dark cycle environment with unlimited access to drinking water and food. All animal experiments were approved by the Ethics Committee of Tongji Medical College.

### 5.3 Weighted gene co‐expression network analysis of HUST platelet proteome dataset and AlzData

The R package WGCNA is used to analyze gene co‐expression networks,^52^, ^53^ including building networks, identifying modules, associating modules with external information, studying module preservation across different data, and finding key drivers in modules of interest.^54^, ^55^ Specifically, the expression matrix includes two expression matrices for all genes and differential genes, and a correlation graph for each module is obtained by clustering according to the expression level of each gene, and then hierarchical clustering tree shows each module, with the gray genes not included in the module. The adjacencies matrix was transformed into topological overlap matrix (TOM), and then the genes were divided into different gene modules based on TOM's difference measure. Here, for the analysis of the overall proteome data, we set the soft threshold power as 9 (scale‐free *R* ^2^ = 0.85), the cut height as 0.25, and the minimum module size as 30 to identify the key modules. Calculating the correlation matrix between traits and genes, only continuous traits can be calculated, and if they are discrete variables, they are converted to a 0–1 matrix when constructing the sample table. The modules of interest were specified for analysis and the genes within the modules were obtained (*p* < 0.05), and the genes highly correlated with traits were also key genes in the trait correlation model.

### 5.4 Differential expression analysis

After normalization of all omics, *t*‐test was used for pairwise comparison analysis (Ctrl vs. MCI; Ctrl vs. AD; MCI vs. AD), *p*‐value <0.05 was considered as significant, and data were expressed as mean ± SEM.

### 5.5 Principal component analysis

The R package FactoMineR was used for PCA to visualize the differences between different groups and samples. Then PCA analysis was performed for the differentially expressed molecules in the platelets. The different groups in the result diagram of all PCA were represented by different colors.

### 5.6 PPI network construction and KEGG analysis

The main steps of analysis include three steps: first, the protein interaction network was obtained from the STRING database, then KEGG enrichment analysis was performed using metascape (https://metascape.org/gp/#/main/),^56^ and finally PPI networks were correlated with the key pathways of enrichment in cytoscape software (version 3.8.2).^57^

First, a PPI network was constructed using STRING database version 11.5 (https://string‐db.org/),^58^, ^59^ selecting “multiple proteins” and uploading a list of protein names, and designating the species under “organism” as *Homo sapiens*. PPI networks consist of nodes representing target proteins and edges representing protein‐protein interactions, and are further analyzed and adjusted in cytoscape software (version 3.8.2). Then, the list of genes was submitted in metascape, the species was selected as *H. sapiens*, and GO and KEGG analyses were performed, and the original data of enrichment results were derived. Finally, the PPI network tab‐separated values (TSV) file was imported from the STRING tool into cytoscape, the node size indicated the link degree, the outer ring color indicated the change in the expression of the corresponding gene in the brain center (TC), and the inner ring color indicated the change in expression of the gene in the peripheral blood (blue indicates downregulation, red indicates upregulation), while orange nodes represent enrichment pathways associated with them.

### 5.7 Analysis of potential targets related to AD pathology

To characterize the association of the dysregulated molecules with the AD‐specific Aβ and tau pathologies, we referred to genome‐wide gene expression analyses and databases related to the development of amyloid or tau pathology,^18^ which were also applied in the AlzData dataset. The genetic symbols of the target proteins were entered into the AlzData dataset, and their correlation with Aβ and tau pathologies was found, and the PPI network of AD and the PPI network connection of AD high‐risk genes (*APP*, *MAPT*, *APOE*, *PSEN1*, and *PSEN2*) was connected.

### 5.8 Machine learning

To evaluate the diagnostic efficiency of potential biomarkers, we employed a classic machine learning approach called cross‐validation with random forests, which was widely used in the field of life sciences to find the best diagnostic model.^10^, ^60^ Specifically, all samples were divided into *k* sample subsets, among which *k* ‐ 1 subsets were taken as the training set and the remaining subset was taken as the validating set. When *k* = *n* (*n* is the number of samples), it is the method of leave‐one‐out cross‐validation: only one sample as the validating set each time, and the other samples are the training set. After *n* cycles, the data can be demonstrated to the maximum extent, and the model obtained is closest to the real result. In order to further demonstrate the reliability of the data, when *k* = 5, it is fivefold cross‐validation: four subsets are taken as the training set each time, and the remaining subset is the validating set, and the mean value is taken to obtain the indicators to determine the diagnostic model.

### 5.9 ELISA and Western blotting validation assays

ELISA kits, ELK1547 and ELK8981 (ELK Biotechnology, Wuhan, China), were used to determine the expression level of HMOX2 in human and mouse plasma, respectively. Western blotting analysis was carried out by following the established procedure by using anti‐HMOX2 primary antibody (1:1000, abcam, ab90492, polyclonal, Rabbit) or anti‐β‐actin primary antibody (1:1000, ABclonal, AC026, monoclonal, Rabbit) or anti‐SERPINA3 primary antibody (1:500, ABclonal, A2803, polyclonal, Rabbit) and horseradish peroxidase (HRP)‐linked secondary antibody (1:3000, Thermo Fisher Scientific, 31460, anti‐Rabbit), and the blots were developed by using a chemiluminescence kit (ECL, Pierce, Thermo Fisher Scientific, 32209).

### 5.10 Immunofluorescence

Brain sections were permeabilized with 0.5% TritonX‐100 in phosphate‐buffered saline (PBS) for 30 min at room temperature, blocked with 5% bovine serum albumin in PBS, and incubated with anti‐HMOX2 primary antibody (1:300, abcam, ab90492, polyclonal, Rabbit) or anti‐SERPINA3 primary antibody (1:200, ABclonal, A2803, polyclonal, Rabbit) for 24 h at 4°C. The second day, the brain sections were washed with 0.1% TritonX‐100 in PBS for three times and incubated with Alexa Fluor 488 secondary antibodies (1:300, Jackson ImmunoResearch, 111‐545‐003, anti‐Rabbit) for 1 h at room temperature. Then, the brain sections were washed with 0.1% TritonX‐100 in PBS for three times and incubated with 4’,6‐diamidino‐2‐phenylindole (DAPI) for 10 min. Finally, brain sections were washed with 0.1% TritonX‐100 in PBS for three times and covered with 50% glycerin in PBS. Pictures were visualized by LSM710 (Zeiss Carl LSM 710, Germany).

### 5.11 Statistical analysis

The student's *t*‐test was used to evaluate the level of significance between the two groups with SPSS 24.0 software (Statistical Program for Social Sciences Inc., Chicago, IL, USA) and GraphPad Prism software 9 (GraphPad Software, Inc., La Jolla, CA, USA). The data were expressed as mean ± SEM and *p*‐values <0.05 were considered to be significant.

## CONFLICT OF INTEREST

The authors declare that they have no conflicts of interest.

## ETHICS STATEMENT

The study was approved by the Tongji Medical School Ethics Committee, complies with the Helsinki Declaration II, and includes written informed consent from all participants. The project “Early Detection of Cognitive Dysfunction in Diabetes” was registered in the Chinese Clinical Trial Registry on April 12, 2013 (https://clinicaltrials.gov; NCT01830998).

## AUTHOR CONTRIBUTIONS

*Experimental design*: H.Y., M.L., Q.P., and J.Z.W. *Experimental methods*: H.Y., M.L., Q.P., Y.L., Y.Z., J.Z.W., T.H., Y.X., Y.W., Y.G., and D.K. *Data analysis*: H.Y., M.L., Q.P., Y.L., and H.Y. *Manuscript writing*: H.Y., M.L., G.C., and J.Z.W. All authors have read and approved the final manuscript.

## Supporting information

Supporting information

## Data Availability

All data used to support the findings of this study are included within the article. Raw data used to generate the figures are available from the corresponding author upon request. Gene expression profiles of large samples of brain regions can be obtained from the AlzData database (http://www.alzdata.org/).
