# Supplementary material for: Integrated analyses of brain and platelet omics reveal their common altered and driven molecules in Alzheimer's disease
Source: MedComm (2020). 2022 Oct 13;3(4):e180. doi: 10.1002/mco2.180 (PMC9560744; doi:10.1002/mco2.180)
Supplement: Supplementary file 1 — Supporting information [file MCO2-3-e180-s001.pdf]

Supplementary materials

Integrated analyses of brain and platelet omics reveal their common altered and driven molecules in Alzheimer's disease

Haitao Yu, Mengzhu Li, Qihang Pan, Yanchao Liu, Yao Zhang, Ting He, Huisheng Yang, Yue Xiao, Ying Weng, Yang Gao, Dan Ke, Gaoshang Chai, Jian-Zhi Wang

Figure. S1 related to Fig. 2. Venn logic analysis flow chart of brain and platelet molecular expression profile. The integration of AlzData datebase and platelet proteomics reflects the coverage depth of platelet proteomics and the statistics of central and peripheral differential molecules.

Figure. S2 related to Fig. 3. Detailed parameters for evaluating the efficiency of biomarkers. (A) Under each best combination, the corresponding protein and various parameters for evaluating the efficiency of the biomarkers. (B) The diagnostic efficiency of the combination biomarkers (IDH3B+RTN1) in the corresponding brain regions and platelets.

Figure. S3 related to Fig. 3. Levels of IDH3B and RTN1 are decreased in both brain and platelet of AD patients. Stars represent significant correlations: *$p < 0.05$; **$p < 0.01$; ***$p < 0.001$; ****$p < 0.0001$.

Figure. S4 related to Fig. 5. Level of HMOX2 (hub molecule) is decreased in both brain of AD patients and mouse models. (A-D) Level of HMOX2 in peripheral system (platelet and plasma) of AD patients and P301L mice. (E) Level of HMOX2 in AD patient brain. (F-G) Level of HMOX2 in P301L mice brain. (H) Level of HMOX2 in hippocampus of 3xTg-AD mice. Stars represent significant correlations: *$p < 0.05$; **$p < 0.01$; ***$p < 0.001$.

Figure. S5 related to Fig. 5. Level of SERPINA3 (hub molecule) is increased in both brain of AD patients and mouse models. (A-B) Level of SERPINA3 in peripheral system (platelet and plasma) of AD patients. (C-F) Level of SERPINA3 in the brain of AD patients. (G-I) Level of SERPINA3 in the brain of 5xFAD, hTau, P301L and 3xTg-AD mice. Stars represent significant correlations: *$p < 0.05$; **$p < 0.01$; ***$p < 0.001$.

Fig. S1 Venn logic analysis flow chart of brain and platelet molecular expression profile.

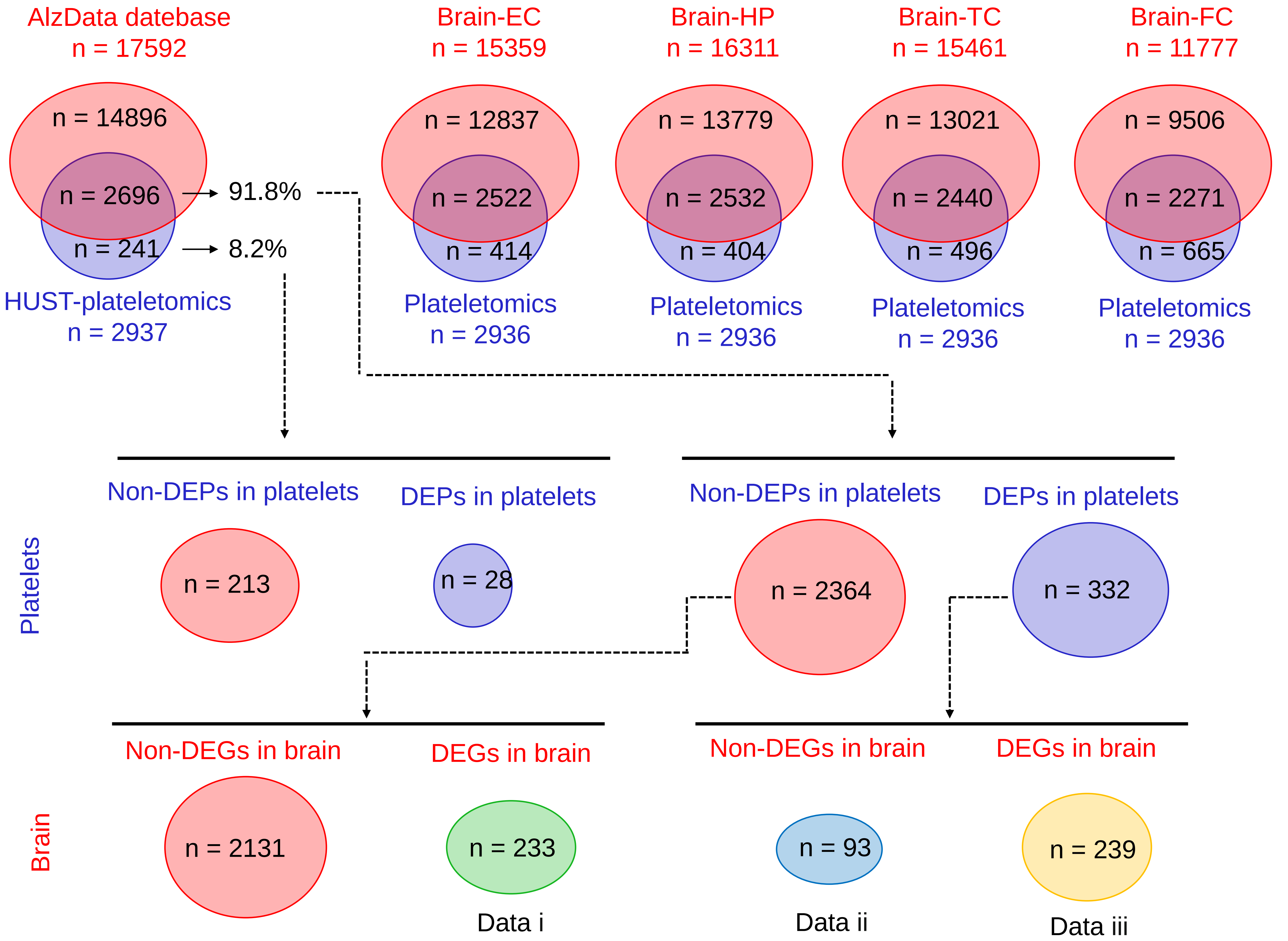

Figure S1 **Yu haitao et al., 2022**

Fig. S2 related to Fig. 3. Detailed parameters for evaluating the efficiency of biomarkers.

A

IDH3B 0	RTN1 1	SRPRA 2	YWHAZ 3	SERPINA3 4	DDAH2 5
**Var**	**AUC**	**F1 score**	**recall**	**precision**	**accuracy**
（0）	0.88	0.84	0.95	0.77	0.75
**（0,1）**	**0.92**	**0.87**	**0.90**	**0.87**	**0.82**
（0,1,3）	0.90	0.84	0.85	0.87	0.78
（0,1,3,4）	0.90	0.81	0.80	0.87	0.75
（0,1,3,4,5）	0.90	0.84	0.80	0.92	0.78
（0,1,2,3,4,5,）	0.90	0.80	0.85	0.78	0.71

B

Var	AUC	F1 score	recall	precision	accuracy
platelet	0.92	0.87	0.90	0.87	0.82
EC	0.86	0.84	0.92	0.77	0.82
TC	0.73	0.71	0.71	0.73	0.67
FC	0.66	0.59	0.60	0.59	0.63
HP	0.66	0.72	0.78	0.68	0.68

Figure S21

Yu haitao et al., 2022

Fig. S3 related to Fig. 3. Levels of IDH3B and RTN1 are decreased in both brain and platelet of AD patients.

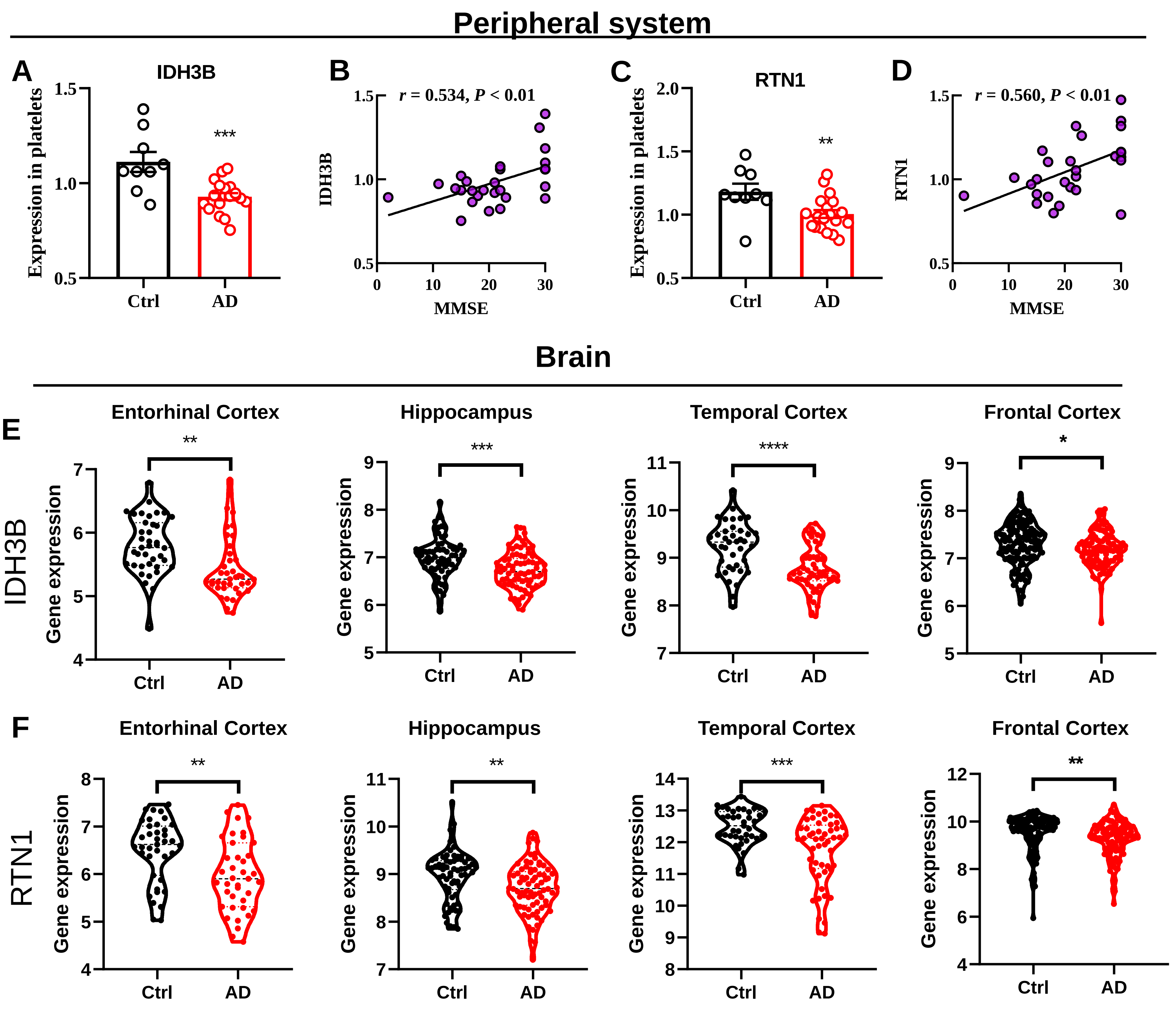

Figure S3

Yu haitao et al., 2022

Fig. S4 related to Fig. 5. Level of HMOX2 (hub molecule) is decreased in both brain of AD patients and mouse models.

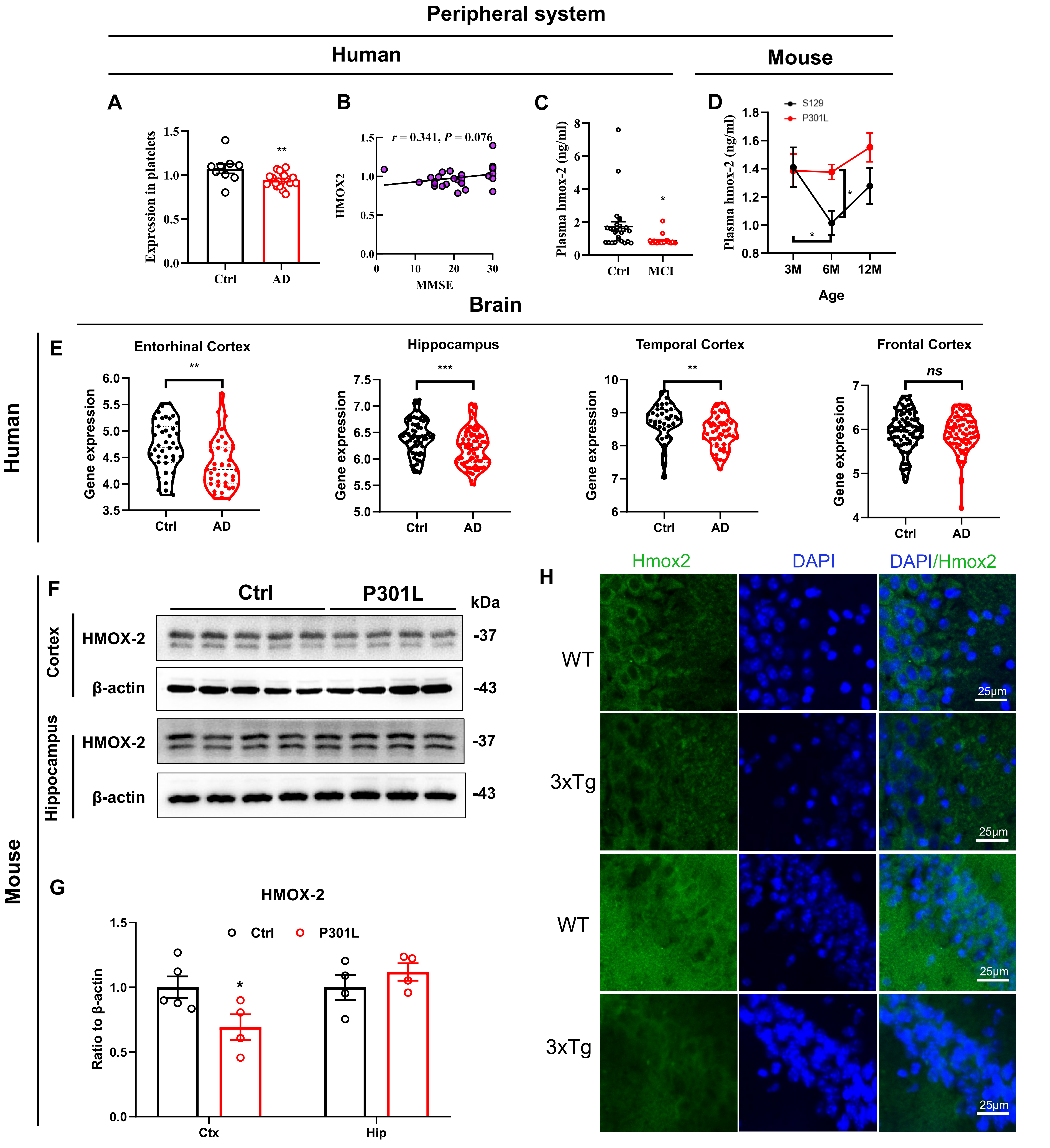

Figure S4

Yu haitao et al., 2022

Fig. S5 related to Fig. 5. Level of SERPINA3 (hub molecule) is increased in both brain of AD patients and mouse models.

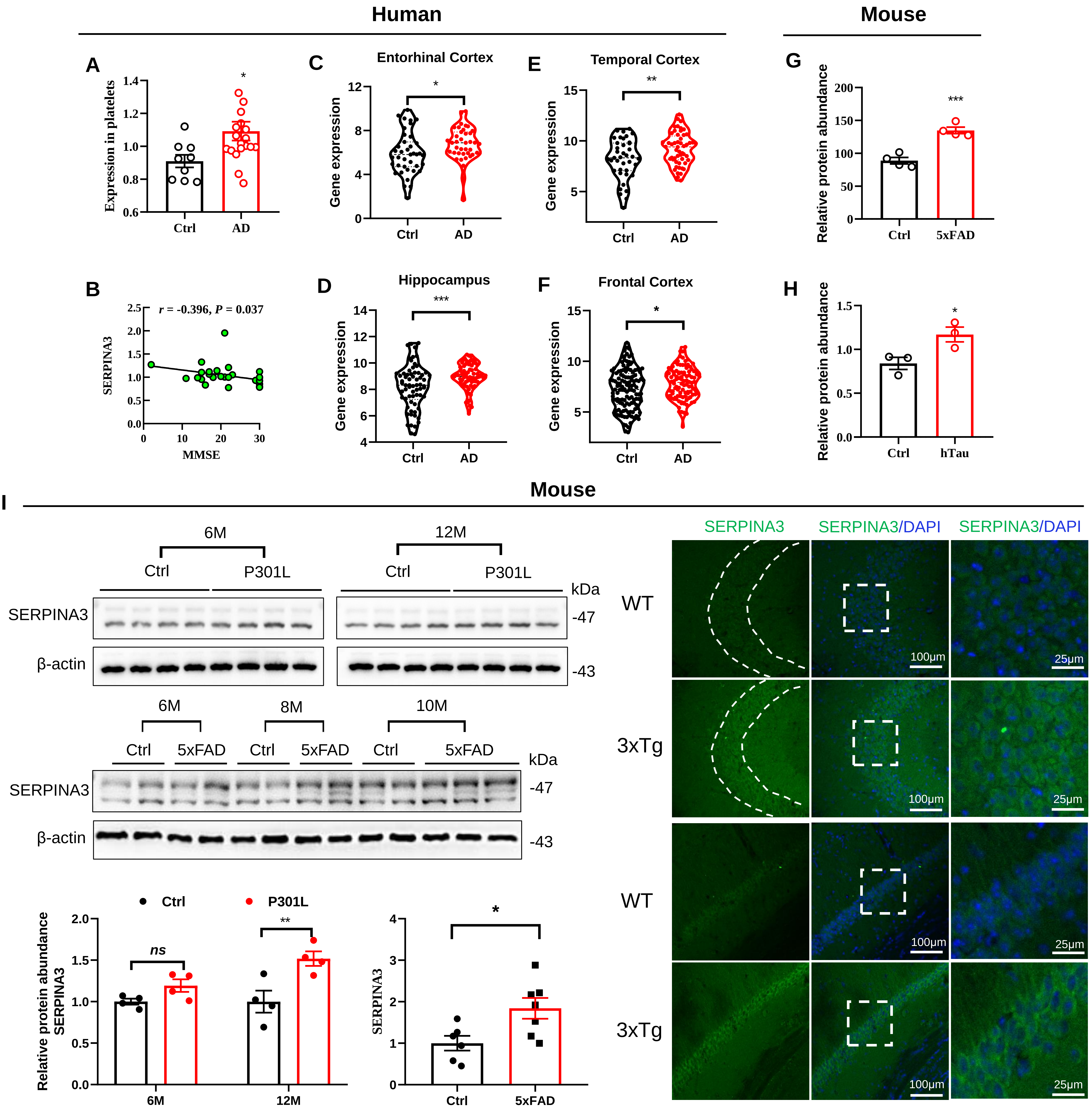

Figure S5

Yu haitao et al., 2022
